# Supplementary material for: CRISPR-Cas9-mediated barcode insertion into Bacillus thuringiensis for surrogate tracking
Source: Microbiol Spectr. 2024 Jul 1;12(8):e00003-24. doi: 10.1128/spectrum.00003-24 (PMC11302227; doi:10.1128/spectrum.00003-24)
Supplement: Supplemental tables — Tables S1-S6. [file spectrum.00003-24-s0001.pdf]

CRISPR-Cas9-mediated barcode insertion into *Bacillus thuringiensis* for surrogate tracking

Supporting Information

Supporting Table 1. Strains and plasmids used in this study.

**Strains/ Plasmids**	**Relevant features**
Bacillus thuringiensis subsp. kurstaki ATCC 33679 (*Btk*)	Strain used for barcode insertion and whole-genome sequencing
MAX Efficiency^{TM} DH5α Competent Cells (*E. coli*)	*E. coli* cells used for plasmid manipulation and triparental mating
E. coli SCS110 Competent Cells	Dam^{-} cells for generating unmethylated DNA
pJOE8999	CRISPR-Cas9 plasmid, Kan^{r}
pJOE8999 sg 183	pJOE8999 vector containing single guide sequence sg 183
pJOE8999 sg183 *Btk* Bn	pJOE8999 vector containing the sg 183 single guide sequence, the *Btk* homology region and respective barcode where n = barcode number (1, 4, 7, 8, or 12)

Supporting Table 2. Barcode sequences and primers used in the present study.

**Name**	**Sequence (5' – 3')**	**[a]Primer sequence (5' – 3')**
B1	GTCACTTCCGCGCAACCTAAATAGAAAAAGAGTTATT GAATTACAAAAAGTATAAAGAAAGCACGACGCGTTAC TCTCCCGGAAAATTTAATTAACTATAGAAATAATTTG CTGTAAAAGTACTGGCGCGGGCTATAAGAAT	F:GTCACTTCCGCGCAACCTAA<br>R:ATTCTTATAGCCCGCGCCAGTA
B4	TGAGACGTCTACCGACTACACTATAAAAATAGTTTCG TAATCAAAAGATTTGAGAATAGGCCGATCACCTTACT AATTCGCCCAGCTGTTCAGAATTAGATATATTATATT ACGTACTGTTAAGACTCCCGGACGATTAGTTATTA	F:TGAGACGTCTACCGACTACACT<br>R:TAATAACTAATCGTCCGGGAGTCT
B7	TACCGATAGGGAGCCTAGTCTATAAATAATACTCCTA TTCTAAACCGAAAAAAATAAATACAGCTCGCCCTTGT GTGACCGGTACTTTCGATACTTTTAATAATTATATTT ACTTCGAGTAGTAACTTCTATCTAGGAATCCGTC	F:TACCGATAGGGAGCCTAGTCT<br>R:GACGGATTCCTAGATAGAAGTTAC
B8	CTTCGTTACGAAACCAGGGACTTGAAAATTTTTTCTT TGAACTTAAGCTTTGATTATCTAACTATCCGACACCC TGACTGTACAGGGTAAACTTGGTACTGAATATATAAG ACTCTAGAAATTAGCAACTAAGTTGTAGAGTAGTCG	F:CTTCGTTACGAAACCAGGGACTT<br>R:CGACTACTCTACAACTTAGTTGCT
B12	CCAGTGTTTCACCCGATTAGCTTTTTTGATTCTGGTT TTTACTTATTTAAGTTAGACCACAAGAACTCGCGTTT GTCTAGAGGGCTATATTTTTAGTTACACAAAGCTCGA ATATTAAAATAGTTCGCTATAAAATACGCCTCAC	F:CCAGTGTTTCACCCGATTAGC<br>R:GTGAGGCGTATTTTATAGCGAACT
sg183	TGAAATGAAATGGTTCAAGT	**/5Phos/**TACGTGAAATGAAATGGTTCAAGT<br>**/5Phos/**AAACACTTGAACCATTTCATTTCA

[a]Bold letters represent a 5' phosphate modification to the primer. Underlined bases are needed as sticky ends for cloning via T4 ligation using the *BsaI* restriction site.

Supporting Table 3. Predicted off-target Cas9 sites from the Cas-OFFinder tool using the *Btk* genome and sg 183 sgRNA sequence 5'-TGAAATGAAATGGTTCAAGTNGG - 3'. Bulge type refers to whether insertions or deletions with respect to the sgRNA were permitted in the off-target analysis search. Bulge size indicates the size of the insertion of deletion permitted (0,1, or 2). If no insertions or deletions were required to generate a significant match, bulge type is reported as 'None' and bulge size is 0.

Bulge type	DNA	Chromosome	Position	Direction	Mismatches	Bulge Size
None	TGAAATGAAATGGTTCAAGTCGG	CP004069.1	1007844	-	0	0
None	TGAAATGAAtcGGTTaAAaTGGG	CP004069.1	2019465	+	4	0
None	cGAAATGAAATGGTTCgAGTAGG	CP004069.1	4219127	-	2	0
None	atAAATcAAATGGTTCAAcTCGG	CP004069.1	4754917	+	4	0
DNA	TAaAAtTaAgATGGTTCAAGTGGG	CP004069.1	1039100	+	4	1
DNA	TAaAAtTaAgATGGTTCAAGTCGG	CP004069.1	1340797	+	4	1
DNA	aTGAAATGAAATGGTTCAAGTCGG	CP004069.1	1007844	-	1	1
DNA	TAaAAtTaAgATGGTTCAAGTCGG	CP004069.1	1106296	-	4	1
DNA	TTGAAATGAAtcGGTTaAAaTGGG	CP004069.1	2019464	+	4	1
DNA	TCGAAAaGAAATGtaTCAAcTGGG	CP004069.1	2594225	+	4	1
DNA	TCGAAATGAAATGGTTCgAGTAGG	CP004069.1	4219127	-	1	1
DNA	aAtAAATcAAATGGTTCAAcTCGG	CP004069.1	4754916	+	4	1
DNA	TGGAgATcAAATGaTTCAAGcTGG	CP004069.1	5251463	-	4	1
DNA	TaAAAtTaAgATGGTTCAAGTGGG	CP004069.1	1039100	+	4	1
DNA	TaAAAtTaAgATGGTTCAAGTCGG	CP004069.1	1340797	+	4	1
DNA	atGAAATGAAATGGTTCAAGTCGG	CP004069.1	1007844	-	2	1
DNA	TaAAAtTaAgATGGTTCAAGTCGG	CP004069.1	1106296	-	4	1
DNA	TGTAAATGAAATaGggCAAaTTGG	CP004069.1	2095186	+	4	1
DNA	TGAAgAaGgAATGGTTgAAGTAGG	CP004069.1	3668093	+	4	1
DNA	TcGAAATGAAATGGTTCgAGTAGG	CP004069.1	4219127	-	2	1
DNA	aaTAAATcAAATGGTTCAAcTCGG	CP004069.1	4754916	+	4	1
DNA	TGGAgATcAAATGaTTCAAGcTGG	CP004069.1	5251463	-	4	1
DNA	TaAAAtTaAgATGGTTCAAGTGGG	CP004069.1	1039100	+	4	1
DNA	TaAAAtTaAgATGGTTCAAGTCGG	CP004069.1	1340797	+	4	1
DNA	atgAAATGAAATGGTTCAAGTCGG	CP004069.1	1007844	-	3	1
DNA	TaAAAtTaAgATGGTTCAAGTCGG	CP004069.1	1106296	-	4	1
DNA	TGATcATGAAATGGTTCgtaTGGG	CP004069.1	1596598	+	4	1
DNA	TGAAgAaGgAATGGTTgAAGTAGG	CP004069.1	3668093	+	4	1
DNA	TcgAAATGAAATGGTTCgAGTAGG	CP004069.1	4219127	-	3	1
DNA	TaAAAtTaAgATGGTTCAAGTGGG	CP004069.1	1039100	+	4	1
DNA	TaAAAtTaAgATGGTTCAAGTCGG	CP004069.1	1340797	+	4	1
DNA	atgAAATGAAATGGTTCAAGTCGG	CP004069.1	1007844	-	3	1
DNA	TaAAAtTaAgATGGTTCAAGTCGG	CP004069.1	1106296	-	4	1
DNA	TGAtCATGAAATGGTTCgtaTGGG	CP004069.1	1596598	+	4	1

DNA	TGAAGtTGAtATGaTTCtAGTTGG	CP004069.1	1689186	+	4	1
DNA	TGAACAaaAAATGGTTCcAaTTGG	CP004069.1	2607506	+	4	1
DNA	TGAAGAaGgAATGGTTgAAGTAGG	CP004069.1	3668093	+	3	1
DNA	TcgAAATGAAATGGTTCgAGTAGG	CP004069.1	4219127	-	3	1
DNA	TGgAGATcAAATGaTTCAAGcTGG	CP004069.1	5251463	-	4	1
DNA	TaAAATTaAgATGGTTCAAGTGGG	CP004069.1	1039100	+	3	1
DNA	TaAAATTaAgATGGTTCAAGTCGG	CP004069.1	1340797	+	3	1
DNA	atgAAATGAAATGGTTCAAGTCGG	CP004069.1	1007844	-	3	1
DNA	TaAAATTaAAgcGGTTCAAGTCGG	CP004069.1	1081839	-	4	1
DNA	TaAAATTaAAgcGGTTCAAGTCGG	CP004069.1	1084763	-	4	1
DNA	TaAAATTaAgATGGTTCAAGTCGG	CP004069.1	1106296	-	3	1
DNA	TGAAgTTGAtATGaTTCtAGTTGG	CP004069.1	1689186	+	4	1
DNA	TGAAgAaGgAATGGTTgAAGTAGG	CP004069.1	3668093	+	4	1
DNA	TcgAAATGAAATGGTTCgAGTAGG	CP004069.1	4219127	-	3	1
DNA	TaAAATTaAgATGGTTCAAGTGGG	CP004069.1	1039100	+	3	1
DNA	TaAAATTaAgATGGTTCAAGTCGG	CP004069.1	1340797	+	3	1
DNA	atgAAaTGAAATGGTTCAAGTCGG	CP004069.1	1007844	-	4	1
DNA	TaAAATTaAAgcGGTTCAAGTCGG	CP004069.1	1081839	-	4	1
DNA	TaAAATTaAgATGGTTCAAGTCGG	CP004069.1	1106296	-	3	1
DNA	TaAAATTaAAgcGGTTCAAGTCGG	CP004069.1	1084763	-	4	1
DNA	TGAAgTTGAtATGaTTCtAGTTGG	CP004069.1	1689186	+	4	1
DNA	TGAAgaAGgAATGGTTgAAGTAGG	CP004069.1	3668093	+	4	1
DNA	TcgAAaTGAAATGGTTCgAGTAGG	CP004069.1	4219127	-	4	1
DNA	TaAAATtAAgATGGTTCAAGTGGG	CP004069.1	1039100	+	3	1
DNA	TaAAATtAAgATGGTTCAAGTCGG	CP004069.1	1340797	+	3	1
DNA	TaAAATtAAAgcGGTTCAAGTCGG	CP004069.1	1081839	-	4	1
DNA	TaAAATtAAAgcGGTTCAAGTCGG	CP004069.1	1084763	-	4	1
DNA	TaAAATtAAgATGGTTCAAGTCGG	CP004069.1	1106296	-	3	1
DNA	TGAAAgGCAAtaaGTTCAAGTAGG	CP004069.1	5468150	+	4	1
DNA	TaAAATtAAgATGGTTCAAGTGGG	CP004069.1	1039100	+	3	1
DNA	TaAAATtAAgATGGTTCAAGTCGG	CP004069.1	1340797	+	3	1
DNA	TaAAATtAAAgcGGTTCAAGTCGG	CP004069.1	1081839	-	4	1
DNA	TaAAATtAAgATGGTTCAAGTCGG	CP004069.1	1106296	-	3	1
DNA	TaAAATtAAAgcGGTTCAAGTCGG	CP004069.1	1084763	-	4	1
DNA	TGcAATGACtAaGGTTCAAGgCGG	CP004069.1	1634511	+	4	1
DNA	TaAAATtAAGATGGTTCAAGTGGG	CP004069.1	1039100	+	2	1
DNA	TaAAATtAAGATGGTTCAAGTCGG	CP004069.1	1340797	+	2	1
DNA	TaAAATtAAAgcGGTTCAAGTCGG	CP004069.1	1081839	-	4	1
DNA	TaAAATtAAAgcGGTTCAAGTCGG	CP004069.1	1084763	-	4	1
DNA	TaAAATtAAGATGGTTCAAGTCGG	CP004069.1	1106296	-	2	1
DNA	TGcAATGAcTAaGGTTCAAGgCGG	CP004069.1	1634511	+	4	1
DNA	TGcAATtAACATGGTTgAAGcAGG	CP004069.1	3951652	-	4	1
DNA	TaAAATtAAgATGGTTCAAGTGGG	CP004069.1	1039100	+	3	1

DNA	TaAAATtAAgATGGTTCAAGTCGG	CP004069.1	1340797	+	3	1
DNA	TaAAATtAAAGcGGTTCAAGTCGG	CP004069.1	1081839	-	3	1
DNA	TaAAATtAAAGcGGTTCAAGTCGG	CP004069.1	1084763	-	3	1
DNA	TaAAATtAAgATGGTTCAAGTCGG	CP004069.1	1106296	-	3	1
DNA	TGAAAgGcAATaaGTTCAAGTAGG	CP004069.1	5468150	+	4	1
DNA	TaAAATtAAgaTGGTTCAAGTGGG	CP004069.1	1039100	+	4	1
DNA	TaAAATtAAgaTGGTTCAAGTCGG	CP004069.1	1340797	+	4	1
DNA	TaAAATtAAAgCGGTTCAAGTCGG	CP004069.1	1081839	-	3	1
DNA	TaAAATtAAAgCGGTTCAAGTCGG	CP004069.1	1084763	-	3	1
DNA	TaAAATtAAgaTGGTTCAAGTCGG	CP004069.1	1106296	-	4	1
DNA	TGtAAaGAAATCtGTTCAAGaTGG	CP004069.1	3046688	-	4	1
DNA	TGAAAgGcAATAaGTTCAAGTAGG	CP004069.1	5468150	+	3	1
DNA	TaAAATtAAAgcGGTTCAAGTCGG	CP004069.1	1081839	-	4	1
DNA	TaAAATtAAAgcGGTTCAAGTCGG	CP004069.1	1084763	-	4	1
DNA	TGtAAaGAAATcTGTTCAAGaTGG	CP004069.1	3046688	-	4	1
DNA	TGAAAgGcAATaAGTTCAAGTAGG	CP004069.1	5468150	+	3	1
DNA	TaAAATtAAAgcGGTTCAAGTCGG	CP004069.1	1081839	-	4	1
DNA	TaAAATtAAAgcGGTTCAAGTCGG	CP004069.1	1084763	-	4	1
DNA	TGAttTGAAATGGCaTCAAtTCGG	CP004069.1	3165217	+	4	1
DNA	TaAAATcAAATGGTTTaAAGaAGG	CP004069.1	4058309	-	4	1
DNA	TGAAAgGcAATaaGTTCAAGTAGG	CP004069.1	5468150	+	4	1
DNA	TGAAtgGAAtTGGTCTaAAGTTGG	CP004069.1	1066683	+	4	1
DNA	TGAttTGAAATGGcATCAAtTCGG	CP004069.1	3165217	+	4	1
DNA	TaAAATcAAATGGTTTaAAGaAGG	CP004069.1	4058309	-	4	1
DNA	TaAAATGAAAcGtTCTaAAGTTGG	CP004069.1	4364147	-	4	1
DNA	TaAAATcAAATGGTTTaAAGaAGG	CP004069.1	4058309	-	4	1
DNA	TGAAgTGAAATGGTTCGAgcgTGG	CP004069.1	2706842	+	4	1
DNA	TaAAATcAAATGGTTtAAAGaAGG	CP004069.1	4058309	-	4	1
DNA	TaAAATcAAATGGTTtAAAGaAGG	CP004069.1	4058309	-	4	1
DNA	TGAAgTGAAATGGTTCgAGcgTGG	CP004069.1	2706842	+	4	1
DNA	TaAAATcAAATGGTTtAAAGaAGG	CP004069.1	4058309	-	4	1
DNA	aGAAAaGAAATtGTTCcAGATTGG	CP004069.1	1291429	+	4	1
DNA	TGAAgTGAAATGGTTCgAGCgTGG	CP004069.1	2706842	+	3	1
DNA	TctAATGAAATGGTaaAAGCTTGG	CP004069.1	3352814	-	4	1
RNA	a-AAtTaAgATGGTTCAAGTGGG	CP004069.1	1039102	+	4	1
RNA	T-AAATGAAATGGTTgcgaTTGG	CP004069.1	998586	+	4	1
RNA	a-AAtTaAgATGGTTCAAGTCGG	CP004069.1	1340799	+	4	1
RNA	a-AAtTaAgATGGTTCAAGTCGG	CP004069.1	1106296	-	4	1
RNA	g-AAATGAAATGGTTCAAGTCGG	CP004069.1	1007844	-	1	1
RNA	T-cAATtAtATGaTTCAAGTCGG	CP004069.1	1815873	+	4	1
RNA	T-AAATGAAATaGggCAAaTTGG	CP004069.1	2095188	+	4	1
RNA	T-cAATGgAAcGcTTCAAGTAGG	CP004069.1	2534545	+	4	1
RNA	T-AgATGAAATtGTTCtAGaAGG	CP004069.1	1437825	-	4	1

RNA	T-AcATGAAtgGGTTCAAaTGGG	CP004069.1	4096766	+	4	1
RNA	g-AAATGAAATGGTTCgAGTAGG	CP004069.1	4219127	-	2	1
RNA	T-AAATcAAATGGTTCAAcTCGG	CP004069.1	4754918	+	2	1
RNA	T-AcAaGAAAcGGTaCAAGTAGG	CP004069.1	5009330	-	4	1
RNA	TG-AAgGAAAcGcTTCAAtTAGG	CP004069.1	771844	+	4	1
RNA	TGA-AgGAAAcGcTTCAAtTAGG	CP004069.1	771844	+	4	1
RNA	TGAA-gGAAAcGcTTCAAtTAGG	CP004069.1	771844	+	4	1
RNA	ga-AATGAAATGGTTCAAGTCGG	CP004069.1	1007844	-	2	1
RNA	gaA-ATGAAATGGTTCAAGTCGG	CP004069.1	1007844	-	2	1
RNA	gaAA-TGAAATGGTTCAAGTCGG	CP004069.1	1007844	-	2	1
RNA	Tc-AATtAtATGaTTCAAGTCGG	CP004069.1	1815873	+	4	1
RNA	TcA-ATtAtATGaTTCAAGTCGG	CP004069.1	1815873	+	4	1
RNA	TcAA-TtAtATGaTTCAAGTCGG	CP004069.1	1815873	+	4	1
RNA	Tc-AATGgAAcGcTTCAAGTAGG	CP004069.1	2534545	+	4	1
RNA	TcA-ATGgAAcGcTTCAAGTAGG	CP004069.1	2534545	+	4	1
RNA	TcAA-TGgAAcGcTTCAAGTAGG	CP004069.1	2534545	+	4	1
RNA	ga-AATGAAATGGTTCgAGTAGG	CP004069.1	4219127	-	3	1
RNA	gaA-ATGAAATGGTTCgAGTAGG	CP004069.1	4219127	-	3	1
RNA	gaAA-TGAAATGGTTCgAGTAGG	CP004069.1	4219127	-	3	1
RNA	Ta-AATcAAATGGTTCAAcTCGG	CP004069.1	4754918	+	3	1
RNA	TaA-ATcAAATGGTTCAAcTCGG	CP004069.1	4754918	+	3	1
RNA	TaAA-TcAAATGGTTCAAcTCGG	CP004069.1	4754918	+	3	1
RNA	TGAAg-GAAAcGcTTCAAtTAGG	CP004069.1	771844	+	4	1
RNA	gaAAt-GAAATGGTTCAAGTCGG	CP004069.1	1007844	-	3	1
RNA	TGAAA-GAgATGGaTaAAGaAGG	CP004069.1	2939144	+	4	1
RNA	TGAAA-GAgAaGtTTCAgGTTGG	CP004069.1	3466948	+	4	1
RNA	gaAAt-GAAATGGTTCgAGTAGG	CP004069.1	4219127	-	4	1
RNA	TaAAt-cAAATGGTTCAAcTCGG	CP004069.1	4754918	+	4	1
RNA	TacAA-GAAAcGGTaCAAGTAGG	CP004069.1	5009330	-	4	1
RNA	TGAAA-GAAtTGGaTaAAaTGGG	CP004069.1	4427636	-	4	1
RNA	TGAAA-GcAATGGTgCAAaaGGG	CP004070.1	64993	+	4	1
RNA	gaAAtg-AAATGGTTCAAGTCGG	CP004069.1	1007844	-	4	1
RNA	TGAAAT-AtATtGaTCAAGaAGG	CP004069.1	1771234	+	4	1
RNA	TcAAtT-AtATGaTTCAAGTCGG	CP004069.1	1815873	+	4	1
RNA	TGgAgT-AAcTGGTTCcAGTGGG	CP004069.1	3544910	-	4	1
RNA	TaAAtc-AAATGGTTCAAcTCGG	CP004069.1	4754918	+	4	1
RNA	TGAAATG-AATctTTCAtGgTGG	CP004069.1	2389193	-	4	1
RNA	TGAAATGA-ATctTTCAtGgTGG	CP004069.1	2389193	-	4	1
RNA	TGAAATGAA-TctTTCAtGgTGG	CP004069.1	2389193	-	4	1
RNA	TGAAAcGAAA-aGcTCAAGaAGG	CP004069.1	1263474	-	4	1
RNA	TGAAATGAgA-GtaTgAAGTTGG	CP004069.1	3891515	-	4	1
RNA	TGAtATGAAAT-caTCgAGTTGG	CP004069.1	162442	+	4	1
RNA	TGAtATGAAATc-aTCgAGTTGG	CP004069.1	162442	+	4	1

RNA	TGAAATGgggT-GTcCAAGTTGG	CP004069.1	1181148	-	4	1
RNA	TGAAATGgggTG-TcCAAGTTGG	CP004069.1	1181148	-	4	1
RNA	TGAAAcGAAAa-GcTCAAGaAGG	CP004069.1	1263474	-	4	1
RNA	TGAAAcGAAAaG-cTCAAGaAGG	CP004069.1	1263474	-	4	1
RNA	TtAAATaAAAT-cTTtAAGTGGG	CP004069.1	5164676	-	4	1
RNA	TtAAATaAAATc-TTtAAGTGGG	CP004069.1	5164676	-	4	1
RNA	TGAtATGAAATca-TCgAGTTGG	CP004069.1	162442	+	4	1
RNA	TGAtATGAAATcaT-CgAGTTGG	CP004069.1	162442	+	4	1
RNA	TtAAAaGAAATaG-TgAAGTAGG	CP004069.1	270740	+	4	1
RNA	TtAAAaGAAATaGT-gAAGTAGG	CP004069.1	270740	+	4	1
RNA	TGAAAcGAAAaGc-TCAAGaAGG	CP004069.1	1263474	-	4	1
RNA	TGAAAcGAAAaGcT-CAAGaAGG	CP004069.1	1263474	-	4	1
RNA	TaAAATGAAATtG-TCAtGaGGG	CP004069.1	2459956	-	4	1
RNA	TaAAATGAAATtGT-CAtGaGGG	CP004069.1	2459956	-	4	1
RNA	TtAAAaGAAATaGTg-AAGTAGG	CP004069.1	270740	+	4	1
RNA	TGAAATGAAtcGGTT-AAaaTGG	CP004069.1	2019465	+	4	1
RNA	TGAAgTGtAATGGaT-AAGcAGG	CP004069.1	1378975	-	4	1
RNA	gGAAATaAAAaGGTT-AgGTTGG	CP004069.1	3884706	-	4	1
RNA	TtAAATaAAATctTT-AAGTGGG	CP004069.1	5164676	-	4	1
RNA	TcAAAcGAAgTGGTTC-AaTAGG	CP004069.1	3031217	-	4	1
RNA	TcAAAcGAAgTGGTTCA-aTAGG	CP004069.1	3031217	-	4	1
RNA	TGAAATGAAtcGGTTaAA-aTGG	CP004069.1	2019465	+	4	1
RNA	TGAAATtAAATGGcTggA-TCGG	CP004069.1	3187832	-	4	1
RNA	TcAAAcGAAgTGGTTCAA-TAGG	CP004069.1	3031217	-	3	1

Supporting Table 4. Editing efficiencies among inserted barcodes in *Btk*

Barcoded Strain	Screened Colonies	Positive Colonies	Editing Efficiency
Btk BAR 1	10	3	30%
Btk BAR 4	18	1	5.6%
Btk BAR 7	10	3	30%
Btk BAR 8	10	6	60%
Btk BAR 12	8	1	12.5%

Supporting Table 5. Alignment information and location of barcode sequences in sequenced *Btk* strains that have been modified by Cas9 to contain a barcode sequence.

Barcode	Percent identity	Alignment length	Mismatches	Gap opens	Subject start	Subject end	E-value
B1	100	164	0	0	1199900	1200063	6.2E-80
B4	100	168	0	0	228931	229098	5.2E-82
B7	100	167	0	0	726068	726234	1.7E-81
B8	100	169	0	0	209954	210122	1.5E-82
B12	100	167	0	0	4606586	4606752	1.7E-81

Supporting Table 6. Frameshift mutations identified in barcoded strains that are not found in *Btk* wild-type strain. Mutations were identified in the re-sequenced genome of the wild-type *Btk* strain used herein by comparing the re-sequenced genome sequence to the reference *Btk* genome sequence. Mutations not shared between the barcoded strains and the Btk reference genome sequence that were not identified in the wild-type *Btk* are reported below.

Strain	Strand	Effect	Product	Coverage	# of strains sharing the mutation
BAR-1	+	frameshift_variant c.380dupA p.Thr128fs	hypothetical protein	192	4
BAR-1	+	frameshift_variant c.453dupT p.Leu152fs	hypothetical protein	193	4
BAR-1	+	frameshift_variant c.135_136insGCTTTGAAAGAAGTTAG p.Ser46fs	ATP-dependent Clp protease proteolytic subunit	48	4
BAR-1	+	frameshift_variant c.925_934delTTTTTTTTTT p.Phe309fs	Histidine kinase	79	4
BAR-1	+	frameshift_variant c.1168_1169insA p.Ser390fs	putative membrane protein ywcF	66	3
BAR-1	+	frameshift_variant c.1165_1166insA p.Ala390fs	3-ketoacyl-CoA thiolase	170	4
BAR-1	-	frameshift_variant c.1513dupA p.Arg505fs	MFS domain-containing protein	134	4
BAR-1	+	frameshift_variant c.383dupT p.Leu128fs	N-acetyltransferase domain-containing protein	135	4
BAR-1	-	frameshift_variant c.590dupA p.Asn198fs	Histidine kinase	32	2
BAR-1	+	frameshift_variant c.598_599insAA p.Val201fs	Abhydrolase-2 domain-containing protein	107	4
BAR-1	+	frameshift_variant c.420delT p.Asn140fs	Response regulator aspartate phosphatase	155	5
BAR-1	+	frameshift_variant&stop_lost&splice_region_variant c.721delT p.Ter241fs	Response regulator aspartate phosphatase	64	4
BAR-1	+	frameshift_variant c.1111_1112insA p.Leu371fs	AI-2E family transporter	73	4
BAR-1	+	frameshift_variant c.804dupA p.Glu269fs	Intein homing endonuclease-related protein	93	4

BAR-1	-	frameshift_variant c.3221dupG p.Ile1075fs	Amino acid adenylation domain-containing protein	115	4
BAR-1	-	frameshift_variant c.1958delG p.Gly653fs	Lipoteichoic acid synthase 1	33	3
BAR-1	+	frameshift_variant c.1479delA p.Lys493fs	putative malate:quinone oxidoreductase	80	4
BAR-1	+	frameshift_variant c.473dupA p.Phe159fs	hypothetical protein	126	4
BAR-1	-	frameshift_variant c.861dupG p.Met288fs	Acetyltransferase	100	4
BAR-1	-	frameshift_variant&missense_variant c.1763delCinsTAG p.Ala588fs	hypothetical protein	24	3
BAR-1	-	frameshift_variant c.593delC p.Pro198fs	ABC transporter substrate-binding protein	106	4
BAR-1	-	frameshift_variant c.796_797delCA p.Gln266fs	DUF2179 domain-containing protein	66	4
BAR-1	-	frameshift_variant c.1047dupA p.Cys350fs	Small ribosomal subunit biogenesis GTPase RsgA	84	4
BAR-1	+	frameshift_variant c.1067_1068delAA p.Lys356fs	PGA-cap domain-containing protein	61	4
BAR-1	+	frameshift_variant c.676dupA p.Ile226fs	hypothetical protein	135	4
BAR-1	+	frameshift_variant c.193_194insA p.Arg65fs	hypothetical protein	175	4
BAR-1	-	frameshift_variant c.247delT p.Cys83fs	hypothetical protein	231	4
BAR-1	-	frameshift_variant c.573delA p.Arg191fs	UvrD-C-2 domain-containing protein	254	2
BAR-1	-	frameshift_variant c.568_569insC p.Leu190fs	UvrD-C-2 domain-containing protein	294	5
BAR-4	+	frameshift_variant c.380dupA p.Thr128fs	hypothetical protein	63	4
BAR-4	+	frameshift_variant c.453dupT p.Leu152fs	hypothetical protein	57	4
BAR-4	+	frameshift_variant c.135_136insGCTTTGAAAGAAGTTAG p.Ser46fs	ATP-dependent Clp protease proteolytic subunit	24	4
BAR-4	+	frameshift_variant c.925_934delTTTTTTTTTT p.Phe309fs	Histidine kinase	43	4
BAR-4	+	frameshift_variant c.1168_1169insA p.Ser390fs	putative membrane protein ywcF	21	3
BAR-4	+	frameshift_variant c.1165_1166insA p.Ala390fs	3-ketoacyl-CoA thiolase	53	4
BAR-4	-	frameshift_variant c.1513dupA p.Arg505fs	MFS domain-containing protein	54	4
BAR-4	+	frameshift_variant c.383dupT p.Leu128fs	N-acetyltransferase domain-containing protein	79	4
BAR-4	+	frameshift_variant c.598_599insAA p.Val201fs	Abhydrolase-2 domain-containing protein	48	4
BAR-4	+	frameshift_variant c.884_896delAAGAAAAAGATAA p.Tyr296fs	hypothetical protein	46	1
BAR-4	+	frameshift_variant c.420delT p.Asn140fs	Response regulator aspartate phosphatase	88	5
BAR-4	+	frameshift_variant&stop_lost&splice_region_variant c.721delT p.Ter241fs	Response regulator aspartate phosphatase	42	4
BAR-4	+	frameshift_variant c.1111_1112insA p.Leu371fs	AI-2E family transporter	45	4
BAR-4	+	frameshift_variant c.804dupA p.Glu269fs	Intein homing endonuclease-related protein	82	4
BAR-4	-	frameshift_variant c.3221dupG p.Ile1075fs	Amino acid adenylation domain-containing protein	76	4
BAR-4	-	frameshift_variant c.1958delG p.Gly653fs	Lipoteichoic acid synthase 1	30	3
BAR-4	+	frameshift_variant c.1479delA p.Lys493fs	putative malate:quinone oxidoreductase	56	4

BAR-4	+	frameshift_variant c.473dupA p.Phe159fs	hypothetical protein	78	4
BAR-4	-	frameshift_variant c.861dupG p.Met288fs	Acetyltransferase	80	4
BAR-4	-	frameshift_variant&missense_variant c.1763delCinsTAG p.Ala588fs	hypothetical protein	20	3
BAR-4	-	frameshift_variant c.593delC p.Pro198fs	ABC transporter substrate-binding protein	98	4
BAR-4	-	frameshift_variant c.796_797delCA p.Gln266fs	DUF2179 domain-containing protein	45	4
BAR-4	-	frameshift_variant c.1047dupA p.Cys350fs	Small ribosomal subunit biogenesis GTPase RsgA	47	4
BAR-4	+	frameshift_variant c.1067_1068delAA p.Lys356fs	PGA-cap domain-containing protein	35	4
BAR-4	+	frameshift_variant c.676dupA p.Ile226fs	hypothetical protein	64	4
BAR-4	+	frameshift_variant c.193_194insA p.Arg65fs	hypothetical protein	60	4
BAR-4	-	frameshift_variant c.247delT p.Cys83fs	hypothetical protein	122	4
BAR-4	-	frameshift_variant c.568_569insC p.Leu190fs	UvrD-C-2 domain-containing protein	171	5
BAR-7	+	frameshift_variant c.380dupA p.Thr128fs	hypothetical protein	77	4
BAR-7	+	frameshift_variant c.453dupT p.Leu152fs	hypothetical protein	85	4
BAR-7	+	frameshift_variant c.135_136insGCTTTGAAAGAAGTTAG p.Ser46fs	ATP-dependent Clp protease proteolytic subunit	22	4
BAR-7	+	frameshift_variant c.925_934delTTTTTTTTTT p.Phe309fs	Histidine kinase	44	4
BAR-7	+	frameshift_variant c.1165_1166insA p.Ala390fs	3-ketoacyl-CoA thiolase	72	4
BAR-7	-	frameshift_variant c.1513dupA p.Arg505fs	MFS domain-containing protein	56	4
BAR-7	+	frameshift_variant c.383dupT p.Leu128fs	N-acetyltransferase domain-containing protein	58	4
BAR-7	+	frameshift_variant c.598_599insAA p.Val201fs	Abhydrolase-2 domain-containing protein	54	4
BAR-7	+	frameshift_variant c.420delT p.Asn140fs	Response regulator aspartate phosphatase	72	5
BAR-7	+	frameshift_variant&stop_lost&splice_region_variant c.721delT p.Ter241fs	Response regulator aspartate phosphatase	27	4
BAR-7	+	frameshift_variant c.1111_1112insA p.Leu371fs	AI-2E family transporter	28	4
BAR-7	+	frameshift_variant c.804dupA p.Glu269fs	Intein homing endonuclease-related protein	51	4
BAR-7	-	frameshift_variant c.3221dupG p.Ile1075fs	Amino acid adenylation domain-containing protein	64	4
BAR-7	+	frameshift_variant c.1479delA p.Lys493fs	putative malate:quinone oxidoreductase	31	4
BAR-7	+	frameshift_variant c.473dupA p.Phe159fs	hypothetical protein	53	4
BAR-7	-	frameshift_variant c.861dupG p.Met288fs	Acetyltransferase	57	4
BAR-7	-	frameshift_variant c.593delC p.Pro198fs	ABC transporter substrate-binding protein	54	4
BAR-7	-	frameshift_variant c.796_797delCA p.Gln266fs	DUF2179 domain-containing protein	29	4
BAR-7	-	frameshift_variant c.1047dupA p.Cys350fs	Small ribosomal subunit biogenesis GTPase RsgA	41	4
BAR-7	+	frameshift_variant c.1067_1068delAA p.Lys356fs	PGA-cap domain-containing protein	32	4
BAR-7	+	frameshift_variant c.676dupA p.Ile226fs	hypothetical protein	66	4

BAR-7	+	frameshift_variant c.193_194insA p.Arg65fs	hypothetical protein	76	4
BAR-7	-	frameshift_variant c.247delT p.Cys83fs	hypothetical protein	122	4
BAR-7	-	frameshift_variant c.573delA p.Arg191fs	UvrD-C-2 domain-containing protein	148	3
BAR-7	-	frameshift_variant c.568_569insC p.Leu190fs	UvrD-C-2 domain-containing protein	174	5
BAR-8	+	frameshift_variant c.380dupA p.Thr128fs	hypothetical protein	108	4
BAR-8	+	frameshift_variant c.453dupT p.Leu152fs	hypothetical protein	83	4
BAR-8	+	frameshift_variant c.135_136insGCTTTGAAAGAAGTTAG p.Ser46fs	ATP-dependent Clp protease proteolytic subunit	28	4
BAR-8	+	frameshift_variant c.925_934delTTTTTTTTTT p.Phe309fs	Histidine kinase	70	4
BAR-8	+	frameshift_variant c.1168_1169insA p.Ser390fs	putative membrane protein ywcF	22	3
BAR-8	+	frameshift_variant c.1165_1166insA p.Ala390fs	3-ketoacyl-CoA thiolase	118	4
BAR-8	-	frameshift_variant c.1513dupA p.Arg505fs	MFS domain-containing protein	106	4
BAR-8	+	frameshift_variant c.383dupT p.Leu128fs	N-acetyltransferase domain-containing protein	114	4
BAR-8	-	frameshift_variant c.590dupA p.Asn198fs	Histidine kinase	23	2
BAR-8	+	frameshift_variant c.598_599insAA p.Val201fs	Abhydrolase-2 domain-containing protein	105	4
BAR-8	+	frameshift_variant c.420delT p.Asn140fs	Response regulator aspartate phosphatase	166	5
BAR-8	+	frameshift_variant&stop_lost&splice_region_variant c.721delT p.Ter241fs	Response regulator aspartate phosphatase	51	4
BAR-8	+	frameshift_variant c.1111_1112insA p.Leu371fs	AI-2E family transporter	93	4
BAR-8	+	frameshift_variant c.804dupA p.Glu269fs	Intein homing endonuclease-related protein	121	4
BAR-8	-	frameshift_variant c.3221dupG p.Ile1075fs	Amino acid adenylation domain-containing protein	140	4
BAR-8	-	frameshift_variant c.1958delG p.Gly653fs	Lipoteichoic acid synthase 1	43	3
BAR-8	+	frameshift_variant c.1479delA p.Lys493fs	putative malate:quinone oxidoreductase	98	4
BAR-8	+	frameshift_variant c.473dupA p.Phe159fs	hypothetical protein	161	4
BAR-8	-	frameshift_variant c.861dupG p.Met288fs	Acetyltransferase	189	4
BAR-8	-	frameshift_variant&missense_variant c.1763delCinsTAG p.Ala588fs	hypothetical protein	37	3
BAR-8	-	frameshift_variant c.593delC p.Pro198fs	ABC transporter substrate-binding protein	126	4
BAR-8	-	frameshift_variant c.796_797delCA p.Gln266fs	DUF2179 domain-containing protein	78	4
BAR-8	-	frameshift_variant c.1047dupA p.Cys350fs	Small ribosomal subunit biogenesis GTPase RsgA	81	4
BAR-8	+	frameshift_variant c.1067_1068delAA p.Lys356fs	PGA-cap domain-containing protein	53	4
BAR-8	+	frameshift_variant c.676dupA p.Ile226fs	hypothetical protein	127	4
BAR-8	+	frameshift_variant c.193_194insA p.Arg65fs	hypothetical protein	124	4
BAR-8	-	frameshift_variant c.247delT p.Cys83fs	hypothetical protein	138	4

BAR-8	-	frameshift_variant c.568_569insC p.Leu190fs	UvrD-C-2 domain-containing protein	232	5
BAR-8	-	frameshift_variant c.593_594insA p.Glu199fs	hypothetical protein	1081	2
BAR-12	+	frameshift_variant c.420delT p.Asn140fs	Response regulator aspartate phosphatase	19	5
BAR-12	-	frameshift_variant c.568_569insC p.Leu190fs	UvrD-C-2 domain-containing protein	34	5
BAR-12	-	frameshift_variant c.593_594insA p.Glu199fs	hypothetical protein	397	2
